# Supplementary figures and images for: Attitudes and practices of open data, preprinting, and peer-review—A cross sectional study on Croatian scientists
Source: PLoS One. 2021 Jun 21;16(6):e0244529. doi: 10.1371/journal.pone.0244529 (PMC8216536; doi:10.1371/journal.pone.0244529)

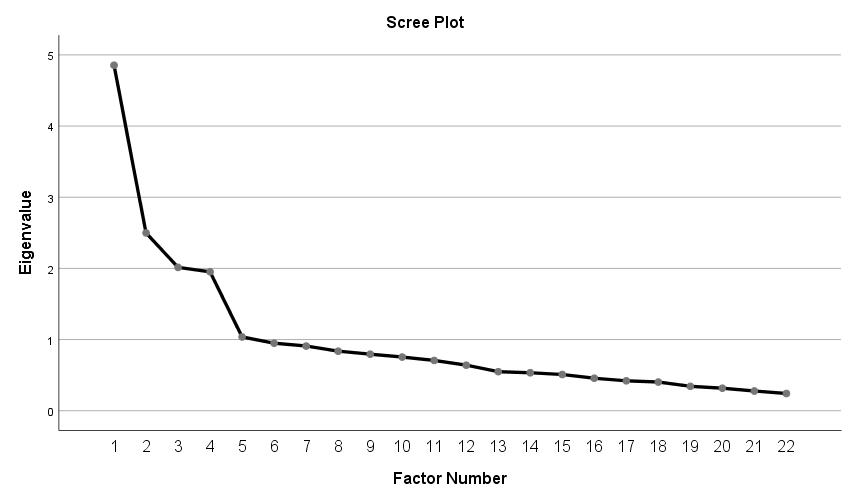

Supplement: S1 Fig — (TIF) [file pone.0244529.s003.tif]
